# Diversity: The Art of Reviewing Independently Together

**DOI:** 10.1523/ENEURO.0350-18.2018

**Published:** 2018-09-11

**Authors:** Christophe Bernard

The theme of this Peer Review Week is diversity, and I paraphrase the original citation of M. Forbes, “Diversity: the art of thinking independently together,” because it says it all. In my last editorial, I addressed the issue of gender balance in the scientific publication process. I showed that *eNeuro* was gender-balanced, i.e., male and female last authors have a similar chance for their submitted paper to be accepted. The same result (gender balance) was found for first authors. I proposed that the double-blind review system was largely contributing to this success. Since the names of authors are not revealed to reviewers, they cannot, ideally, bias their evaluation on gender, country of origin, or reputation/status of the last author.

However, in scientific journals, there is a potential bias that is not frequently taken into consideration, how reviewers are selected. When I acted as a Reviewing Editor for *JNeurosci*, I tried to choose the best reviewers for a given paper, i.e., those who provided excellent factual and fair reviews. I did not pay attention to gender or country of origin of reviewers. Yet, my selection was based on people I knew (and trusted), which I now acknowledge could have introduced a bias in itself; however, I was not cognizant of this potential bias at that time. I would hazard a guess that some of my fellow reviewing editors follow a similar process.

The topic of this Peer Review Week led me to question the way I was doing it. When acting as authors, reviewers, or reviewing editors, we all notice how different reviews can be. We often experience situations in which other reviewers pick up certain scientific issues (caveats, other possible interpretations, etc.) that we failed to notice. This is expected, and this is the diversity reviewing editors hope for to ensure a thorough review.

But there is also a large diversity in the tone used in reviews, from unnecessarily antagonistic or unconstructive to extremely constructive and practical. Many variables influence this tone, including state-of-mind when writing the review (grant deadlines, tiredness, being on vacation, etc.). Much variation in review quality also stems from the reviewer’s psychology, e.g., their persona and experience, how they learned to review papers, and how they consider science (from highly competitive fields to fields where new findings are widely celebrated). As reviewing editors, we can acknowledge and act on “negative” diversity in scientific review, because we know it hinders reaching a fair decision. As *eNeuro* is a Society journal that serves the neuroscience community, we embrace an iterative, accommodating process to scientific review, and I, for one, advocate that adverse and contentious reviews do not have a place at *eNeuro*.

Another form of diversity among scientists stems from their cultural background. This diversity can influence the way experimental science itself is conceptualized. For example, for one reviewer, a major issue in terms of the scientific method used by the authors is only a minor issue for another. I can use my personal experience as a concrete example. French education is strongly influenced by the Cartesian way of thinking. As a result, writing science in French is not the same as writing science in English (a cultural outcome). When I started my career, I was transposing the structure of my reasoning from French to English, which did not work with reviewers. Learning to present science in English was difficult…and still is.

Scientists from a particular cultural background (e.g., Asia) may have a distinct way in which they think about science (something I have noticed in some papers). The same principle can be transposed to the way we review. As a reviewer, my Cartesian mind picked up some failures in logic (from my point of view), which were not mentioned by the other reviewers. But who am I to say that the way I review is better than that of others? I do not. Rather, I think that we should embrace diversity in the way that science is evaluated and accept what others have to say and the way in which they say it. We do not want to reach a thermodynamic equilibrium, a “fit-for-all” state, as this would hinder progress in science. It is a difficult exercise. Reflecting further on my past as a reviewing editor, I wonder whether I unintentionally biased my selection of reviewers towards those who think the same way that I do, because it was more comfortable intellectually. This question is worth considering in more detail, perhaps as a discussion topic for our upcoming *eNeuro* blog site (look for an announcement introducing the blog soon!).

Another interesting issue to consider regarding diversity is the influence of the reviewer’s gender. At *eNeuro*, reviewers do not know who the authors are, so they cannot bias their evaluation on gender considerations. In such “controlled” conditions, it would be interesting to investigate whether female and male reviewers (from similar cultural backgrounds and positions) evaluate manuscripts in the same manner.

As final food for thought, I would like to mention the effect of time. I clearly witnessed a change in the way science has been reported and evaluated over time, at least in my field of electrophysiology. When I started my research career, the reference journal for me was the *Journal of Physiology* (London). In that journal, you would report every single experiment, stating how many results were in line with the hypothesis and how many were not. It seems that accepting variability in experimental outcome was the norm at that time. Being honest about one’s results was part of the scientific publication process, and this was accepted by the reviewers. If you try to do this in a high-profile journal now, you can guess how your paper is going to end up. In high-profile journals, papers are smooth, every experiment works and goes in the same direction to support the conclusions. In my opinion, this is counterproductive because this is not what really happens in biology (cf. the works of Eve Marder). Yet, this is what the reviewers and reviewing editors want from authors for publication in high-profile journals; no edges that do not fit with the general story allowed. *Mea culpa*, when I review for these journals, I also expect a “perfect” story. This attitude has also percolated to many other journals. As such, the way in which science is being evaluated is becoming more standardized and less diverse. Some reviewers may accept that not all results fit with the theory but that this does not constitute a deterrent for publication. Other reviewers may consider that anything that does fit should lead to rejection of the entire study. These are two different ways to approach science. On the same line of thought, you can read published articles on how to present results without *p* values. Questioning the way we do, report, and evaluate science shows that the field is alive and that things change/evolve over time.

In conclusion, I view science in its entirety (experiments, writing a paper, evaluating it, and publishing it) as a complex system. I think that diversity is central and necessary to move forward in improving the scientific review process. As part of the educational outreach efforts at *eNeuro*, we have interactive online training videos about How to Peer Review a Manuscript, aimed at early-career researchers in particular. Perhaps it would be an interesting experiment to assess the review focus of scientists from different cultural backgrounds to encourage all scientists to be more mindful of the opinions of others. I welcome your thoughts and ideas on diversity and inclusion; come join the discussion in this year’s Q&A discussion forum with the SfN Journals’ Editors-in-Chief.

